# Building research capacity in musculoskeletal health: qualitative evaluation of a graduate nurse and allied health professional internship programme

**DOI:** 10.1186/s12913-020-05628-1

**Published:** 2020-08-14

**Authors:** David Wright, Mary Fry, Jo Adams, Catherine Bowen

**Affiliations:** School of Health Sciences, Faculty of Environmental and Life Sciences, University of Southampton, Southampton, SO17 1BJ UK

**Keywords:** Musculoskeletal, Nursing, Allied health professional, Research, Capacity building, Internship, Qualitative

## Abstract

**Background:**

Evidence based practice enhances service planning and delivery, clinical decision making and patient care. However, health professionals often lack the time and opportunity to access or generate evidence. Research capacity building is thus an important mechanism for improving health service delivery. This study evaluates the effectiveness of a UK-wide Nurse and Allied Health Professional musculoskeletal research internship programme in which graduates applied to undertake their internship through one of five Higher Education Institutions. The evaluation explores the experiences of interns and their mentors.

**Methods:**

Sixteen new graduates completed the internship programme (September 2015 – August 2018). Twelve interns and thirteen mentors participated in the evaluation. The evaluation used qualitative asynchronous email-based interviews to explore the experiences of interns and mentors. Interpretive phenomenological analysis of coded transcripts identified principal themes.

**Results:**

Early research outputs from the interns include three peer reviewed publications and 21 conference abstract presentations. Two interns were in full time research at the time of interview or had a research component in their clinical role. Nine interns in clinical posts disclosed plans to return to research in the near future. Seven themes were identified: the impact on interns’ careers; personal impact (for example, influence on self-confidence); impact on clinical practice; drivers for applying; intervention design (for example, attitudes concerning the timing and duration of the intervention); mentorship and networking (including general support provided and quality of career advice); challenges.

**Conclusion:**

The internship programme is an effective model in building research capacity in musculoskeletal research for Nurses and Allied Health Professionals, influencing careers, building confidence and improving clinical practice. The internship programme has the potential to be replicable to other clinical contexts nationally and internationally.

## Background

Building research capacity for healthcare professionals is widely recognised as an important mechanism for enhancing health service delivery [1–3]. Accessing a robust evidence base supports service planning and delivery, informs policy and clinical decision making, and ultimately results in improved patient care [4, 5]. Clinicians play an important role in this process, defining evidence gaps, advising on study design and translating findings into clinical practice, thus enhancing patient outcomes [3]. However, healthcare professionals often lack the opportunity, capacity and skill set capability to access and apply evidence and only a few go on to undertake research [5–7].

Research capacity building seeks to develop research abilities over the long-term, leading ultimately to ‘social change’ within professions [8]. It has had a long-established tradition within medical and dental professions, with early exposure for doctors and dentists to continuous development, dedicated time and opportunities to follow clinical academic careers [1]. However, research capacity building for Nurses and Allied Health Professionals (AHPs) (including physiotherapy, occupational therapy and podiatry) remains significantly underdeveloped in the UK [2, 9]. This is important given the relatively low level evidence for many allied health and nurse interventions and the associated need to build evidence based and informed practice [3–5, 10, 11].

Methods for research capacity building are varied and include single (individuals or within team / organisation) [4] and multiple (across individuals, organisations and policy levels) strategies [12]. They employ various approaches, including partnerships [1, 13], internships [14, 15], bursaries [16], fellowships [17], and mentoring [18]. Evaluations of research capacity models have favoured traditional research outputs metrics, such as number of publications or presentations, although other measures have been assessed [4]. Matus et al., for example, identified the core features that were critical for the success of allied health professional research capacity building frameworks, including supporting clinicians in research, working together and valuing research for excellence [3].

Internships are a recognised model for building research capacity for Nursing and Allied Health Professionals [14, 15]. Previous evaluations have demonstrated that graduate internship schemes for midwifery in Australia [14] and podiatry in the UK [15] have been successful in building research knowledge and experience. Our paper builds on these studies, evaluating an internship programme designed to build research capacity in musculoskeletal (MSK) health for a range of Nursing and Allied Health Professionals in the UK. The aims of this evaluation were to assess the effectiveness of the programme, exploring the experiences of participating interns and mentors and identifying early impact on intern careers and practice.

## Methods

### Programme description

The Nurse and Allied Health Professional musculoskeletal research internship programme ran from September 2015 to August 2018 at five Higher Education Institutions (HEIs) (University of Southampton, University of Leeds, University of Salford, University of Oxford and the University of the West of England Bristol). The aim of the internship was twofold: 1) to provide a foundation for developing AHPs and Nurses as a rheumatology research workforce; 2) to contribute to clinical research and improvement in clinical practice for people living with MSK conditions. Each year, six new graduate podiatrists, physiotherapists, occupational therapists and nurses with a first class or upper second class honours degree were recruited through a competitive process open to all relevant HEIs in the UK. A comprehensive marketing campaign promoted the internship with adverts sent electronically to all UK professional bodies to cascade to undergraduate programme leads and members of professional body organisations. Further promotion occurred through social media (the Versus Arthritis website, intern alumni blogs, Twitter and Facebook) and via programme leads and intern alumni associated with the programme.

**Fig. 1:**
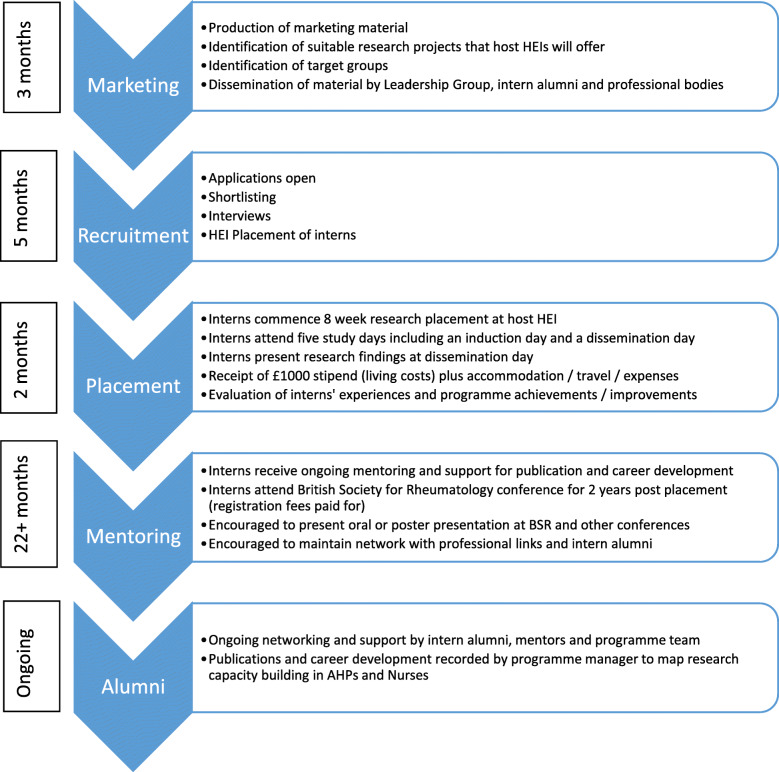
The Nurse and Allied Health Professional Annual Programme Cycle

Eighteen interns were recruited over 3 years. Two withdrew in advance of the internship commencing in 2018 due to personal circumstances, resulting in sixteen interns completing the full programme (Table 1). The internship programme consisted of multiple components (see Fig. 1), including:

- an 8 week intensive research placement at one of the HEIs, during which interns were allocated a research project and mentored by an experienced researcher. Internship projects were diverse, and included a range of methodologies including systematic literature reviews, qualitative methods (e.g. focus groups, semi-structured interviews) and quantitative approaches (e.g. questionnaires). The Leadership Group, comprising representatives from the five HEIs involved, initially generated a long list of topics from which interns could express an interest, matched to their clinical area.
- a 5 day training course delivered over 8 weeks by research leaders, covering MSK and research methods and processes. The course was hosted at each HEI, to which all interns attended.
- 2 years of mentorship by an experienced researcher supporting project completion, paper and abstract preparation, grant writing and general research advice
- a 3 year period of supported networking
- yearly attendance at the British Society of Rheumatology (BSR) annual Conference
- invitation to attend Versus Arthritis’s annual Fellowship Day.

**Table 1:** Number of interns participating by year and profession, and number recruited into the evaluation

Year	Physiotherapist (Recruited)	Podiatrist (Recruited)	Occupational Therapist (Recruited)	Nurse (Recruited)	Total (Recruited)
2016	3 (3)	3 (2)			6 (5)
2017	2 (2)	1 (1)	2 (1)	1 (0)	6 (4)
2018	2 (1)	1 (1)		1 (1)	4 (3)
Total	7 (6)	5 (4)	2 (1)	2 (1)	16 (12)

Each intern was given a £1000 stipend for living expenses and funding for travel and accommodation for attendance at training days hosted by the partner HEI’s and the British Society of Rheumatology annual conference. The structure of the internship was informed by an earlier programme for podiatrists, which ran from 2006 to 2010 [15].

### Study design

The study employed a qualitative outcome evaluation design to assess the impact of the internship programme on Nurse and Allied Health Professional interns and mentors, replicating an approach successfully used to evaluate an earlier programme [15]. Qualitative outcome evaluation has a long history in nursing research [19], and was selected as the qualitative approach enabled a detailed exploration of the lived experience and perceptions of the interns and mentors during and following the internship [20]. A qualitative interpretative phenomenological analysis of asynchronous interviews conducted through email facilitated a rich, detailed understanding of the impact of the internship from the perspective of interns and mentors [21, 22].

### Sample

The evaluation involved a purposive sample of 16 interns and 22 mentors involved in the internship programme. Broad inclusion criteria were used (interns and mentors who completed the internship programme and gave consent to participate). Interns were excluded if they did not complete the internship or were unable or unwilling to give consent or take part in the interviews.

### Participant recruitment

Participants were approached individually by email by the primary investigator (DW). Permission had previously been given by the alumni list owner for the approach to be made. Whilst enhancing ethical standards, an individual approach also highlights the importance of each potential participant and encourages participation [23]. The researcher (DW) employed to evaluate the internship had no previous involvement in the programme and was thus not known to any of the participants. The initial approach to participants introduced both the researcher and his role in the evaluation. Anonymity and confidentiality was assured for all participants. Approval for this study was obtained from the Faculty of Health Sciences, University of Southampton Research Ethics Committee (Ref: ERGO II 41695). All participants gave informed written consent prior to participation.

### Data collection methods

Demographic data were collected from interns at the start of the internship and research output data were collected through email interviews and an annual survey of intern activity. Research outputs were calculated from the time the internship started to data collection, and thus focused solely on internship-related activity.

Email-based semi-structured asynchronous interviews were conducted with interns and mentors to explore experiences of participating in the programme. All interviews were conducted by DW, an experienced male qualitative researcher / evaluator with experience of published qualitative research, evaluations and health service research, who was employed as a research fellow at the time of the evaluation. Email interviews differ from online surveys or virtual focus groups, involving a semi-structured interview conducted between an interviewer and interviewee over an extended time period [21]. Email-based interviews were selected in preference to face-to-face or telephone interviewing primarily due to pragmatic reasons. They cost considerably less to conduct as potential participants were not co-located but were spread across the UK. Conducting email-based interviews thus obviated the need for extensive travel for the investigator and participants. Furthermore, they were the preferred method of data collection for participants due to their adaptability, enabling interns and mentors to respond to questions at a convenient time and location, which was a particular concern for those in full time clinical practice. The potential loss of nuanced data was minimised through the use of acronyms, abbreviations, emoticons and textual emphases. Interviews were conducted using a question schedule informed, in part, by the evaluation of an earlier programme [15]. (See Additional file). Participants received all the primary semi-structured interview questions at the first interview, which took approximately 30 min to complete. Where further reflections or clarifications were required, follow up questions were sent to participants with the responses to previous questions included in the body of the text. This process was repeated up to a maximum of three occasions, enhancing data quality. Fieldnotes of emerging findings were made by the researcher, highlights from which were shared periodically with the co-authors.

### Piloting

The interview method was piloted by the primary investigator (DW) on an individual at the start of the evaluation. Analysis of pilot data involved all members of the evaluation team, and enabled primary questions to be validated, ensuring questions were clear and not open to misinterpretation.

### Data analysis

Interview transcripts were read repeatedly to gain familiarity of the text [22], and were annotated iteratively to identify emerging key concepts. Interpretive phenomenological analysis was undertaken to identify recurring themes [22, 24]. Themes were identified in relation to the prevalence of descriptions, as well as similarities, differences and linguistic connectors [25, 26]. Analyses were conducted by the primary investigator (DW). Academic rigour and trustworthiness of the data were sustained through independent, inter-rater analysis by CB and MF of the pilot and randomly selected transcripts. CB and MF independently identified key themes and an analysis meeting was held with DW at which themes were discussed, degree of agreement was assessed and differences where these occurred were resolved. Analysis occurred iteratively with data collection, with follow-up interviews informed by the results of the early analysis. Verification of the themes from the analysis occurred through the follow-up questions with participants and a presentation of interim and final evaluation findings to a panel comprising mentor and intern representatives. Quotes were extracted from the transcripts to serve as exemplars of identified themes. Participants were identified by a unique code (IT1–12 for interns, ME1–13 for mentors), and all given a female pronoun to protect anonymity. All comments that could potentially identify participants were also removed to ensure anonymity.

## Results

### Participant demographics

Of the sixteen interns who completed the internship programme, twelve participated in the evaluation (3 male, 9 female). One intern was too unwell to take part and three did not respond to the call to participate. The mean age of the interns participating in the evaluation at the start of their internship was 26 years, 1 month (range 20 years, 11 months – 36 years). The graduate profile of the interns was: six physiotherapists, four podiatrists, one occupational therapist and one nurse (Table 1). At the time of interview (September to December 2018), one intern was undertaking a PhD, one had a research component to her clinical role and 10 were in full time clinical positions. Of the 22 mentors approached to participate, 13 took part. Reasons for non-participation of mentors typically related to being too busy or having retired. Of the 13 who took part, there was representation from each of the five HEIs involved, and three participants were male.

All interns participating in the evaluation had presented at least one oral or poster presentation at national / European rheumatology conferences, totalling 21 conference abstracts. Interns also made authorial contributions to three peer reviewed publications, and were lead author for each publication. The number of publications is expected to rise as projects complete: of the nine projects included in the evaluation yet to publish, it is anticipated that at least six will do so. In addition, three interns were successful in grant activity, with funding being awarded for one PhD fellowship, one Clinical Doctoral Research Fellowship, and one MSc.

Analysis identified seven themes, clustered into three domains: 1) impact on careers, interns and practice, 2) drivers for applying, 3) evaluation of the internship design. Each will be described with illustrative quotations.

### Impact of the internship programme

#### Impact on career pathways (the influence of the programme on planned / actual careers)

Mentors and interns valued the programme as it provided an early ‘taster’ for high-achieving graduates who would not otherwise have access to research. As one intern commented, ‘it has allowed people such as myself, recent graduates in healthcare, to gain experience in research which would otherwise not have been possible without pursuing postgrad and research positions (not easy)’ (IT7). Many interns said they gained greater confidence when applying for jobs due to the ‘kudos’ of being an intern and the research and MSK knowledge the internship programme provided: ‘I’ve taken on the responsibility… as a sports physiotherapist, which prior to the internship I would have been apprehensive in committing to due to concerns over a lack of knowledge.’ (IT1).

Most interns said their perceptions of research had changed as a result of the programme. There was increased awareness that clinical academic careers were possible and not harmful to clinical careers, that research was accessible and not ‘elitist’, and that research was clinically relevant and not removed from practice: ‘I learnt what a clinical research career really looked like – working with patient partners and a wide clinical research team to improve patient care. Not just sitting in an office evaluating what has already been done’ (IT3).

The programme was a ‘game changer’ for interns entering research positions, providing valuable knowledge of the research process, greater familiarity of research environments, and an awareness of multiple pathways for research funding. For the ten interns in full time clinical roles, nine said they intended to continue with research in the near future. Furthermore, interns entering clinical roles reported that the MSK knowledge provided by the internship supported their subsequent career.

#### Personal impact (the influence of the programme on self-confidence and personal effectiveness)

Interns reported a growth in confidence as a result of the programme: ‘I grew confidence in myself and my ability to achieve and overcome challenges through having ownership of my project and needing to solve problems and project manage myself’ (IT12). Interns also gained confidence through presenting research and networking with academics and clinicians. They also commented that the programme improved their personal effectiveness, resulting in an ability to manage workloads and to communicate effectively and confidently with colleagues: ‘I have been able to use skills that I feel I developed during my internship, such as critical thinking, problem solving, project management, time management and the ability to communicate with staff and my seniors’ (IT12).

#### Impact on clinical practice (evidence of improved clinical activities)

Interns entering clinical posts described how the programme improved clinical activities through an awareness of evidence based practice and an ability to access relevant information. One intern explained, ‘I am better able to evaluate research and incorporate it into my practice… When looking into creating an injury prevention exercise program for athletes, I used evidence to guide the structure and content, being able to run quick effective searches and quickly sift through endless articles and find the good pieces of evidence’ (IT5). Interns similarly described an improved ability to undertake Quality Improvement and audit, and a deeper understanding of patient-centred care, enhancing the appropriateness of treatments offered: ‘my exposure to service users was really valuable and complemented my clinical experience of trying to make treatments patient centred… I am more aware of ensuring that my treatments are patient centred in design and execution’ (IT5).

The increased confidence noted earlier also improved practice, with interns being able to question and clarify diagnoses and treatment options: ‘I am a lot more confident in my ability to take on responsibility and communicate effectively with others… I’m not afraid to speak with members of the MDT to share my knowledge’ (IT1). Interns also said the knowledge gained of rheumatological symptoms, diagnoses and treatments had improved their clinical practice. As one mentor described, ‘even those interns that do not end up working in rheumatology, the internship gives them essential skills that they can use in practice and become “champions” for rheumatology in their respective work’ (ME5).

### Drivers for applying (rationale for interns applying to the programme)

Principal drivers for applying to the internship programme included an interest in research, a desire to attain a deeper understanding of research processes, and a wish to gain a ‘flavour’ of academia whilst considering career options. As one intern described, ‘I knew I was unable to embark on a clinical academic pathway straight from university due to financial constraints at the time, and the internship was a great option. I got to experience what a real-life research environment was like’ (IT4).

Interns believed that research and clinical knowledge gained through the internship would strengthen their subsequent careers. A knowledge of research processes, such as ethics applications and quantitative / qualitative skills, was thought to be helpful for those wishing to apply for academic / clinical academic posts. Similarly, an understanding of MSK health was thought to be helpful for those wishing to further their clinical careers. Furthermore, interns hoped a deeper understanding of evidence based practice would enhance their clinical activities.

All intern mentors agreed that the application process had been successful in recruiting interns with a commitment and capability for MSK research: ‘those who make it onto the internship programme are driven and ambitious and have already developed a thirst for research for what it can bring to clinical practice’ (ME7).

### Evaluation of internship design

#### Design of the Intervention (assessment of the appropriateness of the programme’s design)

All interns and most mentors said the timing (post-graduation and pre-first post) and duration (8 weeks) of the placement was optimal, resulting in an internship that was ‘intense’ and ‘ambitious’, yet feasible. It was suggested that fewer than 8 weeks would not provide sufficient breadth of experience and would make project deliverables unachievable. Conversely, extending the placement could deter graduates from applying as this would delay the start of clinical / academic posts: ‘eight weeks was a manageable amount of time – any longer may have put me off applying and you wouldn’t be able to get the full internship experience if it were shorter’ (IT3).

Most interns valued the programme’s training days and said they provided an effective introduction to career development, research skills and MSK health. Visiting sites were highly valued as they provided an experience of research and clinical expertise at each location whilst strengthening a sense of intern ‘community’. Most interns welcomed the opportunity to work in research teams, providing an insight into the ‘lived reality’ of research life.

Leading a research project to completion was important to interns, and thus the selection of research projects was integral to the programme’s success. The internship was most rewarding where intern involvement was meaningful: ‘I didn’t feel like a data monkey. I was there to learn and grow’ (IT9). Mentors and interns recognised the challenge of identifying topics that were sufficiently ambitious yet achievable within the timeframe of the programme. Failing to identify a suitably ambitious project would have limited the value of the internship experience.

#### Mentorship and networking (assessment of the quality of mentor and network support)

The programme provided formal mentoring from experienced health researchers and facilitated peer support from interns. Most interns valued the support provided by mentors, particularly their advice on career pathways: ‘mentorship and continued support during and after my internship was absolutely pivotal in allowing me to progress my clinical research career’ (IT3). Mentors reported that hosting interns was a significant investment of time, although all had the experience and capacity to support the programme.

It was evident that the interns developed an effective, supportive community during the programme. As one mentor put it, ‘do not underestimate the power of the peer support network, not just within the eight-week placement, but way beyond’ (ME8). Interns spoke of the ‘lifelong’, ‘invaluable’ and ‘encouraging’ support provided by fellow interns, enabling challenges, opportunities and aspirations to be shared whilst motivating those participating in the programme. The distinction between intern and mentor began to erode over time with previous interns becoming ‘ambassadors’, actively promoting the programme and supporting new graduates. A challenge for the programme was thus providing effective mechanisms for long-term networking.

#### Challenges and areas for improvement (intern and mentor reflections on the programme and recommendations for further development)

Involving 22 mentors across five sites provided a wide range of research and clinical expertise. However, it became apparent that not all shared a common understanding of the programme’s philosophy. Whilst most mentors understood the purpose to be, ‘about offering opportunities and developing the next generation’ (ME2), a minority thought it was primarily focused on research skill development. There is a need therefore to ensure consistent communications are given to mentors.

A challenge identified by interns and mentors was maintaining research interest and completing research activities whilst in full time clinical positions. Interns in clinical roles typically reported a lack of research interest from their clinical teams. As one mentor explained, ‘As newly qualified personnel, their clinical lives are incredibly busy, but also their employers are keen for them to focus on consolidating and learning new clinical skills, so they are very reluctant to free them up for regular mentor sessions, speaking at intern events or moving into higher degrees’ (M6). Consequently, interns spoke of their frustration at being unable to continue with research: ‘I would love to continue research as I am really passionate about it and I enjoy it, however I am not sure how this fits in with working full time and progressing clinically’ (IT8). Interns spoke of feeling ‘anxious’ and pressured as research tasks had to be undertaken outside their clinical work. As one mentor described, ‘the reality of clinical practice can scupper the best laid plans for future work on an incomplete project’ (ME6). Solutions proposed included the careful selection of research projects, increased mentor support, and close engagement with future clinical host organisations.

Another challenge identified by mentors was an underrepresentation from occupational therapy and nursing applicants. One mentor commented on the need for a, ‘targeted promotion to nursing students who constitute the majority of the rheumatology workforce but are minimally represented in the internship programme’ (ME5). A particular problem in attracting nurse graduates was the incompatibility of the timing of the programme with cycles of graduation at some HEIs, with the effect that some nurses would already be in employment by the time the internship started. Solutions to this problem included improved marketing and use of social media and running two cycles of the programme each year.

## Discussion

This study reports findings from the first evaluation of a multi-disciplinary internship programme. It demonstrates that the internship programme is an effective model for research capacity building for Nurses and Allied Health Professionals in MSK health. Quantitative metrics indicate that the internship projects are achieving research impact in terms of numbers of publications and presentations. An evaluation of an earlier podiatry internship, which informed the development of the 2015–18 internship programme, reported a higher number of outputs [15], principally due to metrics being collated over a longer time period, thus indicating that long term outputs will increase.

However, quantitative metrics, though often favoured [4], are not necessarily the most appropriate means for evaluating a programme of this nature. Of greater concern is the extent to which the programme met its original aims, for which a qualitative approach exploring the experiences of interns and mentors is more appropriate. This indicates that the internship has met its first aim, developing Nurses and Allied Health Professionals as a MSK research workforce: interns reported growing confidence, enhanced research skills, greater MSK knowledge and an appreciation that research was not an elitist enterprise. As a result, all but one intern was either involved in research or had plans for further research in the near future. The extent to which the internship programme provided early exposure to research is interesting given that all interns should have undertaken research as part of their undergraduate degrees. Findings from our evaluation therefore indicate that the research content of professional bodies’ educational programmes may require reviewing.

The evaluation also indicates that the internship has met its second aim, contributing to clinical research and improvement in clinical practice: interns continue to complete and disseminate their research and clinical practice was enhanced through an understanding of evidence based practice, patient centred care and improved MSK knowledge.

Our findings reflect published evaluations of similar research capacity building activities. Hauck et al. [14], for example, reported an increase in research awareness, confidence and skills and a desire to use research to inform clinical practice in midwives participating in an Australian graduate internship programme. Clough et al. [17] reported that supporting early career clinical and research training through fellowships increased the likelihood of securing externally funded doctoral training awards with many fellowship-holders moving into academic roles. Naidoo et al. [15] similarly found in their evaluation of a graduate podiatry internship (which was the forerunner of the 2015–18 programme), that the programme was valuable to the interns’ careers and supported research capacity building in foot and ankle rheumatology. They also identified the importance of mentors and peer support, and the impact the internship had on changing perceptions of research [15]. More broadly, our evaluation reflects the core principles for research capacity building identified in the wider internship literature, namely the importance of developing skills and confidence, fostering partnerships and collaborations, and valuing research for excellence [3, 27].

Whilst the internship has been successful, the challenges identified need to be addressed. In particular, the challenge of supporting research activity in a clinical environment that often devalues such enterprises has long be recognised, particularly for Nurses and Allied Health Professionals [28]. Internships adopting a similar model must consider how the transition from internship to clinical practice is best supported. Professional bodies and agencies, such as the Council for Allied Health Professions Research in the UK, have an important role in supporting those who wish to continue research following capacity building activities but find they are unable to do so due to a lack of research support in clinical environments. Given that nearly all the interns continued to use research to inform their practice, it is hoped that they will continue to inculcate a research-positive environment within their teams as they progress to senior management positions.

### Limitations

The evaluation findings were strengthened by the appointment of an independent evaluator (DW) and the assured anonymity of interns and mentors that participated. However, there were several limitations that should be noted. The study employed email interviews as this was the preferred means of approach by study participants. However, a limitation of email interviewing is the loss of non-verbal cues that may be identified in face-to-face interviews and a potential loss of data richness. We overcame this to some extent through the use of acronyms, abbreviations, emoticons and textual emphases. Responses were not obtained from four interns and the two interns who withdrew from the programme were not approached (although they withdrew before the internship had started). The interviews were undertaken on completion of the whole scheme rather than individual internship experiences, and responses may have been influenced by subsequent career activities and recall bias. Several of the mentors participating in the evaluation were also on the Leadership Group responsible for the oversight of the internship, thus representing potential bias in the study findings. However, there were no discernible differences between their views and those of other participants, supported in part by the assured confidentiality of their input. Finally, it is recognised that the time-lag between data collection and the end of the internship was very short (1 month for the 2018 cohort). Whilst this was helpful in terms of memory recall for the eight-week placement and the training days, it was not possible to identify longer-term impact.

## Conclusion and implications of the study

Academic career pathways and evidence based practice for Nurses and Allied Health Professionals remain underdeveloped. Research capacity building is an important mechanism that develops much needed research knowledge, skills and opportunities. The graduate internship programme is an effective model in building research capacity in MSK research for Nurses and Allied Health Professionals, influencing careers, building confidence and contributing to improving clinical practice. Long-term, it is hoped the internship will serve as a secure foundation for a generation of research-aware and research active professionals, thus facilitating the ‘social change’ that is urgently needed in the professions involved. For this to happen, health care institutions have a role in recognising and nurturing the expertise gained through the programme. By evaluating and sharing the impact of the internship, we hope that other professions will consider the value of replicating the model within their own clinical context.

## Supplementary information

**Additional file 1. ** Evaluation Question Schedules

## Data Availability

Due to the qualitative nature of the evaluation and the ethical considerations given, it is not possible to share data from this study.

## Abbreviations

AHP: Allied Health Professional
ERGO: Ethics and Research Governance Online
MDT: Multidisciplinary Team
MSK: Musculoskeletal
