# Supplementary material for: Building research capacity in musculoskeletal health: qualitative evaluation of a graduate nurse and allied health professional internship programme
Source: BMC Health Serv Res. 2020 Aug 14;20:751. doi: 10.1186/s12913-020-05628-1 (PMC7429677; doi:10.1186/s12913-020-05628-1)
Supplement: Supplementary file 1 — Additional file 1. Evaluation Question Schedules [file 12913_2020_5628_MOESM1_ESM.docx]

**Additional File: Evaluation Question Schedules**

***Questions sent to interns:***

1. What year did your internship take place?

2. What made you choose to apply for the Arthritis Research UK graduate internship?

3. What are your views on the structure of the internship?

4. What are your views on the internship overall?

5. What were the key things that you learned from the internship?

6. Have you been able to apply the skills you learned during the internship to your current role? If yes, please explain how? If no, please explain why?

7. Did your perception of research change as a result of taking part in the internship? Please explain your response.

8. Did your perception of rheumatology change as a result of taking part in the internship? Please explain your response.

9. Did your perception of yourself change as a result of taking part in the internship? Please explain your response.

10.What are your views on the mentorship and continued support during and post-internship?

11. How do you feel now that the internship programme has ended?

12. Can you tell me what you have been doing since completing the internship?

13. What are your plans for your future career?

14. What changes, if any, would you suggest be made to future internship programmes?

***Questions sent to mentors:***

1. Do you think the Arthritis Research UK graduate internship programme for AHPs and Nurses has been successful? Please explain your response.

2. What are your views on the application and selection process for the internship?

3. What are your views on the structure of the internship?

4. What are your views on the internship overall?

5. What were the key things that you learned from the internship?

6. How do you feel now that the internship has ended?

7. What changes, if any, would you suggest be made to future internship programmes?
